# Decrease in rate of multiple sclerosis-related hospitalizations in Portugal

**DOI:** 10.12688/f1000research.8787.1

**Published:** 2016-06-13

**Authors:** Marta Pereira, Dimitra Lambrelli, Sreeram V. Ramagopalan

**Affiliations:** 1 Real-World Evidence, Evidera, London, W6 8DL, UK

**Keywords:** multiple sclerosis, epidemiology, hospitalizations

## Abstract

We sought to investigate the rate of multiple sclerosis (MS)-related hospitalizations in Portugal and assess whether there have been temporal changes as described in other countries. Using data from the Portuguese National Discharge Registry, we observed that between 2008 and 2013 the rate of MS-related hospitalizations decreased by 44%, from 15.9/100 person-years (95% confidence interval (CI: 14.9-16.9) in 2008 to 8.9/100 person-years (95% CI: 8.2-9.6) in 2013. The change in hospitalization rates is in accordance with what has been observed in other countries, and coincides with the release of new therapies for MS in Portugal.

## Introduction

Multiple sclerosis (MS) is a chronic, neurodegenerative autoimmune disorder of the central nervous system ^1^. With a prevalence of 1/800 in North America and Northern Europe, MS is the most common acquired neurological disorder in young adults ^1^, posing a substantialburden on healthcare systems. Recent studies have, however, suggested that MS-related hospitalization rates have been declining over the last decades ^2, 3^.

Portugal is considered a region of medium MS prevalence ^4^, but epidemiological data are limited. In the present study, we sought to investigate the difference in the rate of MS-related hospitalizations in Portugal between 2008 and 2013.

## Methods

Data on the number of hospitalizations in 2008 and 2013 with an MS diagnosis [International Classification of Diseases, 9th revision (ICD 9): 340] were obtained from the National Hospital Discharge Registry, centrally held in the Portuguese Central Administration of the Health System. This database includes data about all hospitalizations in all public hospitals. In Portugal, the National Health Service provides universal access to healthcare and patients with MS are almost exclusively treated in public hospitals ^4^. These years were chosen as MS prevalence data in 2008 and 2013 in Portugal were available from the Atlas of MS ( http://www.msif.org/wp-content/uploads/2014/09/Atlas-of-MS.pdf) ^5^. The total number of hospitalizations (1,177,048 in 2008 and 1,108,911 in 2013), as well as the estimates of the Portuguese population, were obtained from Portuguese official statistics ^6^.

The incidence of hospitalizations (MS-related or all-cause hospitalizations) was calculated by dividing the number of hospitalizations in each year by the number of patients at risk in the same year. The Wald method was used to calculate 95% confidence intervals (CI). Two different definitions of MS-related hospitalizations were used: 1) where MS was the primary reason for admission — the MS ICD-9 diagnostic code was reported in the field of the primary diagnosis, and 2) where MS was reported as either a primary or a secondary reason for admission — the MS ICD 9 diagnostic code was in any position on the diagnoses fields (20 fields available). The proportion of MS-related hospitalizations in each year was obtained by dividing the number of MS-related hospitalizations in each year by the total number of hospitalizations in the same year. Demographic and clinical characteristics of patients with MS hospitalized in each year were described using number and percentage of patients for categorical variables, and mean and standard deviation (variables with a normal distribution) or median and interquartile range (variables with a skewed distribution) for continuous variables. Differences between characteristics in 2008 and 2013 were estimated using t-tests (variables with a normal distribution) or Mann–Whitney test (variables with a skewed distribution) for continuous variables, and Chi square test for other categorical variables. P values < 0.05 were considered to be statistically significant. Statistical analysis was performed using STATA software version 13.

## Results

**Figure 1.**
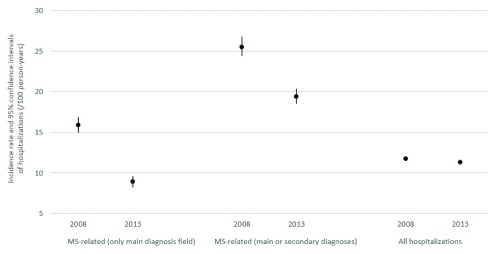
Incidence rate of MS-related and all hospitalizations in Portugal in 2008 and 2013.

Demographic and clinical characteristics of patients with MS hospitalized in 2008 and 2013 are summarized in Table 1. Between 2008 and 2013, the incidence rate of MS-related hospitalizations decreased from 15.9/100 person-years (95% CI: 14.9–16.9) to 8.9/100 person-years (95% CI: 8.2–9.6), defined using only information recorded in the main diagnosis field, and from 25.5/100 person-years (95% CI: 24.4–26.8) to 19.4/100 person-years (95% CI: 18.5–20.4) using the main or secondary diagnoses respectively ( Figure 1). In the same years, the incidence rate of all hospitalizations in Portugal decreased from 11.7/100 person-years (95% CI: 11.7–11.7) to 11.2/100 person-years (95% CI: 11.1–11.2) ( Figure 1). The proportion of MS-related hospitalizations among all hospitalizations in Portugal decreased slightly from 0.07% to 0.05% between 2008 and 2013.

**Table 1.** Demographic and clinical characteristics of patients with MS hospitalized in 2008 and 2013.

	MS as main diagnosis			MS as main or secondary diagnosis		
	2008 (N=795)	2013 (N=576)	P value	2008 (N=1277)	2013 (N=1264)	P value
**Age of patients** **hospitalized, years**						
Mean	39.1	40.5	0.033	42.8	46.5	<0.001
SD	11.9	12.3		13.4	14.6	
**Gender, n (%)**						
Male	264 (33.2)	164 (28.5)	0.062	457 (35.8)	387 (30.6)	0.006
Female	531 (66.8)	412 (71.5)		820 (64.2)	877 (69.4)	
**Length of stay (days)**						
Median	3	4	0.006	4	4	<0.001
IQR	1–6	1–6		1–7	2–8	
**Deaths during** **hospitalization, n (%)**	7 (0.9)	2 (0.4)	0.227	39 (3.0)	35 (2.8)	0.669
**Other diagnosis during** hospitalization, n (%)*						
Infectious and parasitic diseases	3 (0.4)	3 (0.5)	0.691	25 (2.0)	22 (1.7)	0.685
Neoplasms	7 (0.9)	9 (1.6)	0.246	62 (4.9)	74 (5.8)	0.263
Endocrine, nutritional and metabolic diseases and immunity disorders	93 (11.7)	112 (19.4)	<0.001	188 (14.7)	325 (25.7)	<0.001
Diseases of the blood and blood-forming organs	21 (2.6)	24 (4.2)	0.118	63 (4.9)	122 (9.6)	<0.001
Mental disorders	68 (8.6)	130 (22.6)	<0.001	136 (10.6)	277 (21.9)	<0.001
Diseases of the nervous system and sense organs (excluding MS)	347 (27.5)	154 (26.7)	<0.001	202 (15.8)	117 (14.7)	<0.001
Diseases of the circulatory system	60 (7.6)	106 (18.4)	<0.001	201 (15.7)	370 (29.3)	<0.001
Diseases of the respiratory system	24 (3.0)	23 (4.0)	0.328	126 (9.9)	180 (14.2)	0.001
Diseases of the digestive system	11 (1.4)	18 (3.1)	0.027	68 (5.3)	139 (11.0)	<0.001
Diseases of the genitourinary system	49 (6.2)	48 (8.3)	0.112	179 (14.0)	297 (23.5)	<0.001
Diseases of the skin and subcutaneous tissue	10 (1.3)	9 (1.6)	0.634	35 (2.7)	72 (5.7)	<0.001
Diseases of the musculoskeletal system and connective tissue	20 (2.5)	29 (5.0)	0.013	47 (3.7)	90 (7.1)	<0.001
Congenital anomalies	2 (0.2)	0 (0.0)	0.228	4 (0.3)	6 (0.5)	0.516
Symptoms, signs, and ill-defined conditions	43 (5.4)	118 (20.5)	<0.001	92 (7.2)	233 (18.4)	<0.001
Injury and poisoning	6 (0.8)	7 (1.2)	0.385	58 (4.5)	112 (8.9)	<0.001

* Described according to the groups of diseases defined in the International Classification of Diseases 9 ^th^ revision MS, multiple sclerosis; SD, standard deviation; IQR, interquartile range

The age of the patients with MS hospitalized and the length of stay in the hospital increased significantly ( Table 1) between 2008 and 2013, where MS was either only a primary diagnosis or a diagnosis anywhere on the patient record. There was an increase in the proportions of females admitted to hospital but this was only significant when MS was recorded in any of the diagnosis fields ( Table 1; p value = 0.062 for MS as main diagnosis and p value = 0.006 for MS as main or secondary diagnosis).

No differences were observed in the proportion of patients that died during hospitalization ( Table 1). Where MS diagnosis was anywhere on the patient record, the proportion of MS patients with other diagnoses during hospitalization increased significantly between 2008 and 2013 for all groups of diseases considered, except for infectious and parasitic diseases, neoplasms, diseases of the nervous system and sense organs (excluding MS), and congenital anomalies ( Table 1).

Data of multiple sclerosis related hospitalizations in Portugal The raw data of incidence rate of multiple sclerosis related hospitalization between 2008 and 2013 are reported. Click here for additional data file. Copyright: © 2016 Pereira M et al. 2016 Data associated with the article are available under the terms of the Creative Commons Zero "No rights reserved" data waiver (CC0 1.0 Public domain dedication).

## Discussion

Here we show a decrease in MS-related hospitalizations in Portugal from 2008 to 2013. Where MS was the primary diagnosis the decrease was substantial — approximately 44%. The rates of hospitalization observed in Portugal appear to be similar to that documented in Canada ^2, 3^, suggesting perhaps that the thresholds for admission are similar between the two countries. The change in hospitalization rates cannot be explained entirely by a general change in admissions in Portugal, although this did decline by 4%. The change in hospitalization coincides with the release of new therapies for MS, the first of which, natalizumab (Tysabri ^®^), became available in June 2007 in Portugal ^7^. It is plausible that these newer therapies prevented some hospital admissions for patients with MS. The average age of MS patients admitted in 2013 was older as compared to 2008. As MS prevalence has increased over the period, the decrease in hospitalization rate may reflect a diluting by more newly diagnosed patients with lower disease severity. There may also be a change in the way healthcare is delivered, with a possible shift to more outpatient services as seen in other countries. This may explain why the average length of stay for MS-related admissions has increased while the rate of admissions has decreased, although this change in disease management has generally preceded the time period we have investigated in other countries.

Limitations of this study include the lack of clinical data and information on potential confounders. There are also the uncertainties associated with the prevalence data we had available to us. Despite these limitations, our results are in accordance with previous results on hospital admissions in patients with MS. Our findings provide further epidemiological data on MS in Portugal, healthcare resource use in these patients, and impetus to investigate other efforts to reduce hospitalizations in this population.

## Data availability

The data referenced by this article are under copyright with the following copyright statement: Copyright: © 2016 Pereira M et al.

Data associated with the article are available under the terms of the Creative Commons Zero "No rights reserved" data waiver (CC0 1.0 Public domain dedication). 

The Portuguese Central Administration of the Health System provided the database and gave permission to use the data to describe in the article. The data are not available online, but are available upon request after approval of the study objectives.

F1000Research: Dataset 1. Data of multiple sclerosis related hospitalizations in Portugal, http://dx.doi.org/10.5256/f1000research.8787.d125589 ^8^
