# Supplementary material for: Decrease in rate of multiple sclerosis-related hospitalizations in Portugal
Source: F1000Res. 2016 Jun 13;5:1353. [Version 1] doi: 10.12688/f1000research.8787.1 (PMC4937828; doi:10.12688/f1000research.8787.1)
Supplement: Peer review history

# Peer review: Referee response for version 1

**DOI:** 10.5256/f1000research.9457.r14810

**Published:** 2016-07-06

**Authors:** Bruce Taylor

**Affiliations:** 1 Menzies Research Institute Tasmania, University of Tasmania, Hobart, Australia

This is an interesting short report that documents a significant fall in the number of hospitalisations for MS in Portugal following the release of higher potency disease modifying drugs. The observation is interesting and adds some support to the presumed greater efficacy of these therapies. Although causality cannot be determined from such an observational study.

 The finding that the age of admissions increased is also interesting as it may indicate that those with longer duration of disease (directly correlated with age) may have acquired more complications of MS for example urosepsis and have required greater admission rates. 

 To directly answer the question significantly more detail and prospective acquisition of data is required. however this report does provide a strong background rationale to undertake these studies and to acquire this type of data which is invaluable in assessing the benefits or otherwise of expensive and potentially higher risk therapies in MS.

I have read this submission. I believe that I have an appropriate level of expertise to confirm that it is of an acceptable scientific standard.

# Peer review: Referee response for version 1

**DOI:** 10.5256/f1000research.9457.r14457

**Published:** 2016-06-21

**Authors:** João Correia de Sa

**Affiliations:** 1 Department of Neurosciences and Mental Health, Santa Maria University Hospital - North Lisbon Hospital Center, Lisbon, Portugal

The paper by Pereira and co-workers addresses an relevant issue: to bring to public some data about the hospitalization rates of MS patients in Portugal.

 There are no methodological errors but, in my opinion, some changes should be introduced:

1. About Introduction when the authors say that the epidemiological data in Portugal are limited I can´t agree. Portugal is a small country of about 10 million people and 3 prevalence studies and 1 incidence study published bring data enough for a knowledge of the basic epidemiological picture of MS in the country. There is of course other missing data (for instance mortality data/ burden of the disease for the society) but these are often missing in other countries. I considered changing saying for instance that though prevalence and incidence in MS in Portugal are now known, other epidemiological data about the disease are still lacking.
2. The author stay that they have found a "decrease in MS related hospitalizations..... from 2008 to 2013". In fact they have only these two dates for comparison. They have no data among these two years so that some variability can not be excluded.
3. In Portugal there are a great diversity of neurological care. Central Hospitals in Lisbon, Oporto and Coimbra treat MS in ambulatory but in other Towns: Évora,Beja, Leiria, Covilhã, Santarem, for example patients are admitted for treating relapses with Methyl, and for Natalizumab infusion because Day-Hospital units are not available. This should be taken in consideration in discussion and could certainly explain why there are so many short-time admissions

I have read this submission. I believe that I have an appropriate level of expertise to confirm that it is of an acceptable scientific standard.
